# The feasibility of automated online flow cytometry for *in-situ* monitoring of microbial dynamics in aquatic ecosystems

**DOI:** 10.3389/fmicb.2014.00265

**Published:** 2014-06-02

**Authors:** Michael D. Besmer, David G. Weissbrodt, Bradley E. Kratochvil, Jürg A. Sigrist, Mathias S. Weyland, Frederik Hammes

**Affiliations:** ^1^Department of Environmental Microbiology, Eawag – Swiss Federal Institute for Aquatic Science and TechnologyDübendorf, Switzerland; ^2^Department of Environmental Systems Science, Institute of Biogeochemistry and Pollutant Dynamics, ETH ZürichZürich, Switzerland; ^3^Institute of Environmental Engineering, Chair of Process Engineering in Urban Water Management, ETH ZürichZürich, Switzerland

**Keywords:** flow cytometry (FCM), bacteria, drinking water, monitoring, sensors, automation, online, staining

## Abstract

Fluorescent staining coupled with flow cytometry (FCM) is often used for the monitoring, quantification and characterization of bacteria in engineered and environmental aquatic ecosystems including seawater, freshwater, drinking water, wastewater, and industrial bioreactors. However, infrequent grab sampling hampers accurate characterization and subsequent understanding of microbial dynamics in all of these ecosystems. A logic technological progression is high throughput and full automation of the sampling, staining, measurement, and data analysis steps. Here we assess the feasibility and applicability of automated FCM by means of actual data sets produced with prototype instrumentation. As proof-of-concept we demonstrate examples of microbial dynamics in (i) flowing tap water from a municipal drinking water supply network and (ii) river water from a small creek subject to two rainfall events. In both cases, automated measurements were done at 15-min intervals during 12–14 consecutive days, yielding more than 1000 individual data points for each ecosystem. The extensive data sets derived from the automated measurements allowed for the establishment of baseline data for each ecosystem, as well as for the recognition of daily variations and specific events that would most likely be missed (or miss-characterized) by infrequent sampling. In addition, the online FCM data from the river water was combined and correlated with online measurements of abiotic parameters, showing considerable potential for a better understanding of cause-and-effect relationships in aquatic ecosystems. Although several challenges remain, the successful operation of an automated online FCM system and the basic interpretation of the resulting data sets represent a breakthrough toward the eventual establishment of fully automated online microbiological monitoring technologies.

## Introduction

Monitoring microbial dynamics in engineered and environmental aquatic ecosystems is a key step toward a better understanding of the driving forces and consequences of changes in bacterial concentrations and community composition (Stadler et al., 2008; Read et al., 2011). Such ecosystems are often highly dynamic. For instance, engineered ecosystems such as drinking water production and wastewater treatment plants follow daily routines in operation, may suffer from short-term malfunctioning, and are exposed to dynamic changes in raw water composition (Gunther et al., 2012; Hwang et al., 2012). Environmental ecosystems such as oceans, lakes, and groundwater are subject to daily or seasonal variations or sudden events (e.g., acute pollution) (Guadayol et al., 2009; Stadler et al., 2010). Bacterial parameters tend to respond to such variations and events. To establish the links between the microbial dynamics and their causes and implications, accurate and high-throughput measurements of microbial parameters are necessary (Van Den Hoven et al., 2009). Specifically, in the field of drinking water, an improved understanding of microbial dynamics can support process optimization, microbial risk assessment, and risk management (e.g., Smeets et al., 2010, World Health Organization, 2011). When investigating this type of dynamics, it is crucial to collect data for sufficiently long periods and at high sampling frequencies to fully capture the respective type of pattern (e.g., daily, weekly) (Stadler et al., 2008). This makes optimal monitoring a challenge with respect to the choice of method(s) and practical *in-situ* implementation.

Only a limited number of microbial detection methods are suitable for monitoring aquatic ecosystems in the detailed manner described above. Major constraints are low sensitivity for changes, low reproducibility, and high demand for labor, time and other resources (Sheikh et al., 2012). In view of these constraints, flow cytometry (FCM) is a promising method for high-throughput routine measurements (Porter et al., 1997; Vives-Rego et al., 2000; Czechowska et al., 2008; Hammes and Egli, 2010) and hence has been widely applied in the analysis of ecosystems including seawater (Legendre et al., 2001), freshwater (Gregori et al., 2001), wastewater (Foladori et al., 2010), and drinking water (Vital et al., 2012). Direct FCM measurements are possible for organisms with auto-fluorescence (e.g., phytoplankton) (Pomati et al., 2013) or organisms with fluorescence reporter genes [e.g., green fluorescence protein (GFP)] (Arnoldini et al., 2013). Other microorganisms—such as small, environmental bacteria—need to be stained with a fluorescent dye before measurement in order to distinguish them from background and/or to detect specific cellular features (Prest et al., 2013). In light of the requirements for ecosystem monitoring discussed above, one of the principle challenges is to develop robust, automated FCM systems that can stain and measure over extended time periods (e.g., weeks to months) in short intervals (e.g., 10–15 min).

The concept of FCM automation is not new. The first automated systems with and without staining procedures were developed for medical and biotechnology applications (Omann et al., 1985; Lindberg et al., 1993; Zhao et al., 1999; Abu-Absi et al., 2003). In environmental sciences, automated systems were developed for auto-fluorescent pigments (e.g., phytoplankton) (Dubelaar et al., 1999; Thyssen et al., 2008). More recently systems were described for measuring fluorescence from genetically modified organisms in biotechnology (Broger et al., 2011; Brognaux et al., 2013) and a prototype was developed specifically for drinking water analysis (Hammes et al., 2012). The latter study describes laboratory-scale testing of automated sampling, staining, incubation, and measurement as an interesting technique for monitoring aquatic ecosystems. Full-scale application and implementation of automated FCM, though coming with considerable technological and scientific challenges, offer immense potential. Until now automated FCM studies that include fluorescent staining of cells have been limited to short-term (<60 h) experiments for industrial processes or research applications. Long-term automated FCM measurements focusing on monitoring environmental ecosystems have not yet been explored and considered in depth.

We developed a new prototype online FCM system capable of meeting these challenges. Here we demonstrate the application of this instrumentation and approach on two aquatic ecosystems (drinking water and river water) of differing microbiological quality and dynamics. The novelties of this study are: (i) *in-situ* analysis of real water samples subject to natural events (e.g., rainfall), (ii) fully automated analysis during extended time periods (days-to-weeks), and (iii) detailed analysis of large data sets with respect to microbiological baselines, patterns, and correlations with abiotic sensor data. In addition we critically discuss the advantages and challenges of online FCM and consider the added value of such large data sets in a variety of applications.

## Materials and methods

### Online FCM system

A fully automated staining robot was constructed based on the principles of a previously described system (Hammes et al., 2012). In short, the system was designed to collect discrete water samples at 15-min intervals from a given source (e.g., flowing tap, flowing river). Upon collection the sample was mixed at a 1:1 ratio with a solution of fluorescent dye [SYBR Green I (Life Technologies, Eugene OR, USA)] diluted in sterile TRIS buffer (10 mM, pH 8.0). The final concentration of SYBR Green I in the sample relative to the stock solution was 1:10,000. A bacteriostatic concentrate solution (BD Accuri, Ann Arbor MI, USA) was added to the dye at 5 μL mL^−1^ to avoid contamination in the stain solution. The sample-dye mixture was subsequently incubated for 10 min at 40°C and afterwards automatically pumped to, and analyzed with, a C6 flow cytometer (BD Accuri, San Jose CA, USA). The C6 flow cytometer measured at a flow rate of 66 μL min^−1^ for 30 s with a lower threshold on the green fluorescence (FL1-H) set at 1000. All other Accuri C6 flow cytometer specifications, filters, and measured variables were identical to those described in Prest et al. (2013). All tubing, pumping, and mixing chambers were rinsed with nanopure water between sampling. The sampling line was flushed with the sample water prior to every new measurement to avoid contamination from earlier steps and ensure that a fresh sample was collected. A cleaning cycle with hypochlorite (1% active chlorine), detergent (Partec, Goerlitz, Germany), and nanopure water was automatically initiated every 24 h.

### Engineered ecosystem: drinking water

Drinking water samples were drawn at 15-min intervals from a continuously flowing cold water tap (16.0 ± 0.5°C, 0.5 L min^−1^) in the Eawag laboratory building (Dübendorf, Switzerland) during an experimental period of 14 days. The tap water was non-chlorinated groundwater supplied as drinking water by the local water utility. No other parameters were measured during the course of this experiment, but the same water was described in detail in a previous study (Lautenschlager et al., 2010).

### Environmental ecosystem: river water

A shallow, oligotrophic river (Chriesbach, Dübendorf, Switzerland) was monitored during 12 consecutive days. Samples were measured with online FCM at 15-min intervals from a sedimentation basin, located in an experimental facility adjacent to the river, into which the river water was continuously pumped (approximate retention time: 30 min). The same water was simultaneously fed to a 14-L-PVC reactor (at 0.9 L min^−1^) where four abiotic parameters [temperature (T), dissolved oxygen (DO), electrical conductivity (EC) and pH] were measured with online sensor probes (Table 1) once every 10 s in parallel to the FCM measurements. The DO, EC, and pH sensors were automatically compensated for temperature changes.

**Table 1:** **Overview of measured parameters and employed measurement devices for each water type**.

**Measured parameters**	**Tap water**	**River water**	**Measurement device**	**Figure/Table**
Total cell concentration	x	x	Online FCM	Figures 1–6
Percentage of LNA content bacteria	x	x	Online FCM	Figures 1–6
Conductivity		x	Online sensor	Figures 5, 6
Temperature		x	Online sensor	Figures 5, 6
pH		x	Online sensor	Figures 5, 6
Oxygen concentration		x	Online sensor	Figures 5, 6
Rainfall volume		x	Weather station [NABEL (FOEN and EMPA)]	Figures 3, 5

### Data extraction and analysis

**Figure 1:**
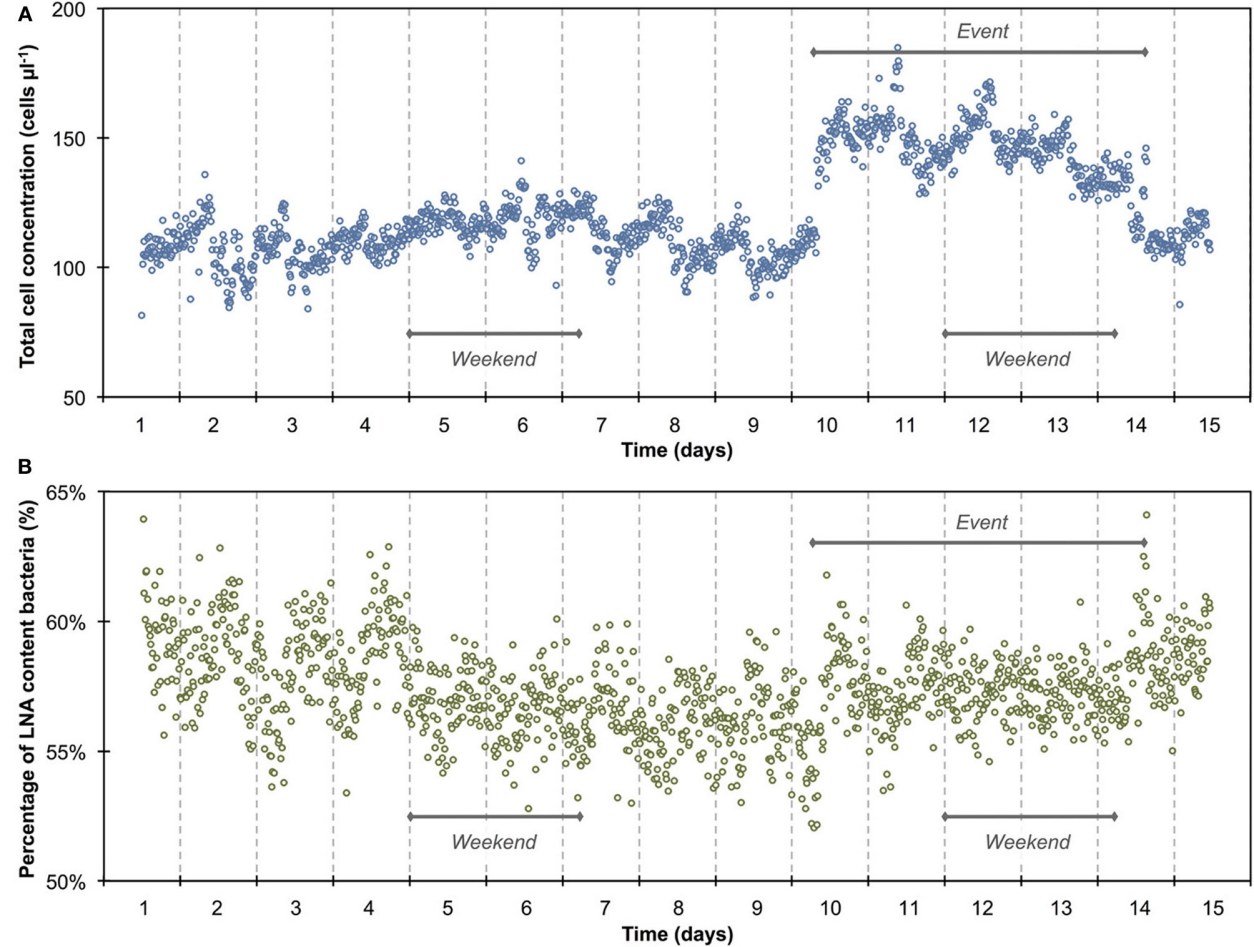
**Online FCM measurements of continuously flowing municipal tap water over 14 days, showing the total cell concentration (TCC) (A) and percentage of LNA content bacteria (B) (*n* = 1302)**. Data points are single measurements at 15-min intervals of water samples stained with SYBR Green I. Daily patterns can be identified for both parameters. In addition, a distinct event with a 50% increase in TCC can be seen from day 10 to day 14 whereas the percentage of LNA content bacteria was barely affected.

The raw FCM data files were analyzed with custom software that enabled batch processing of the large data sets generated in this study. In short, FCM gates were constructed to separate signals of stained bacterial cells from signals of the background and distinguish between the so-called high (HNA) and low (LNA) nucleic acid content bacterial cells. The gating strategy was based on descriptions provided elsewhere (SLMB, 2012; Prest et al., 2013). All generated data, i.e., total cell concentration (TCC) and LNA content bacteria concentration, were subsequently exported in csv format for final processing in MS Excel. In order to separately analyze regular daily patterns and specific events, subsets were extracted from both data sets by subjective selection. Both the data selection and the reasoning behind it are discussed and illustrated in detail in the results and discussion sections below, in Figures 1, 3, and in Table S1 (Supplementary information). The online sensor data set for abiotic parameters was reduced to one point every 15 min to match the sampling frequency of the online FCM. Multivariate analyses were computed in R (R Development Core Team, 2008) according to a numerical ecology methodology analogous to the one developed (Weissbrodt et al., 2014) based on Borcard et al. (2011). In short, pair-wise x-y plots were computed between the average baselines of all FCM and sensor parameters for a first visual observation of any apparent correlations. The pair-wise correlations between the full baselines of all parameters were then quantified by the computation of Pearson's correlation coefficients (PCC, linear relationship) and Spearman's rank correlation coefficients (monotonic relationship) after standardization of the FCM and sensor data sets (see Section 8 in supplementary information). The significance of the correlations was assessed by the computation of *p*-values at 95% confidence level. The pair-wise coefficients were displayed in a heat map for efficient representation of the gradients in positive and inverse correlations between parameters, and for rapid identification of the pre-dominant correlations. In this heat map, the parameters were reordered by hierarchical clustering using the Ward algorithm (see Section 8 in supplementary information). The additional R packages Vegan (Oksanen et al., 2009), Heatplus (Ploner, 2011), and Heatmap.plus (Day, 2007) were used to these ends.

## Results

### Engineered ecosystem: drinking water

**Figure 2:**
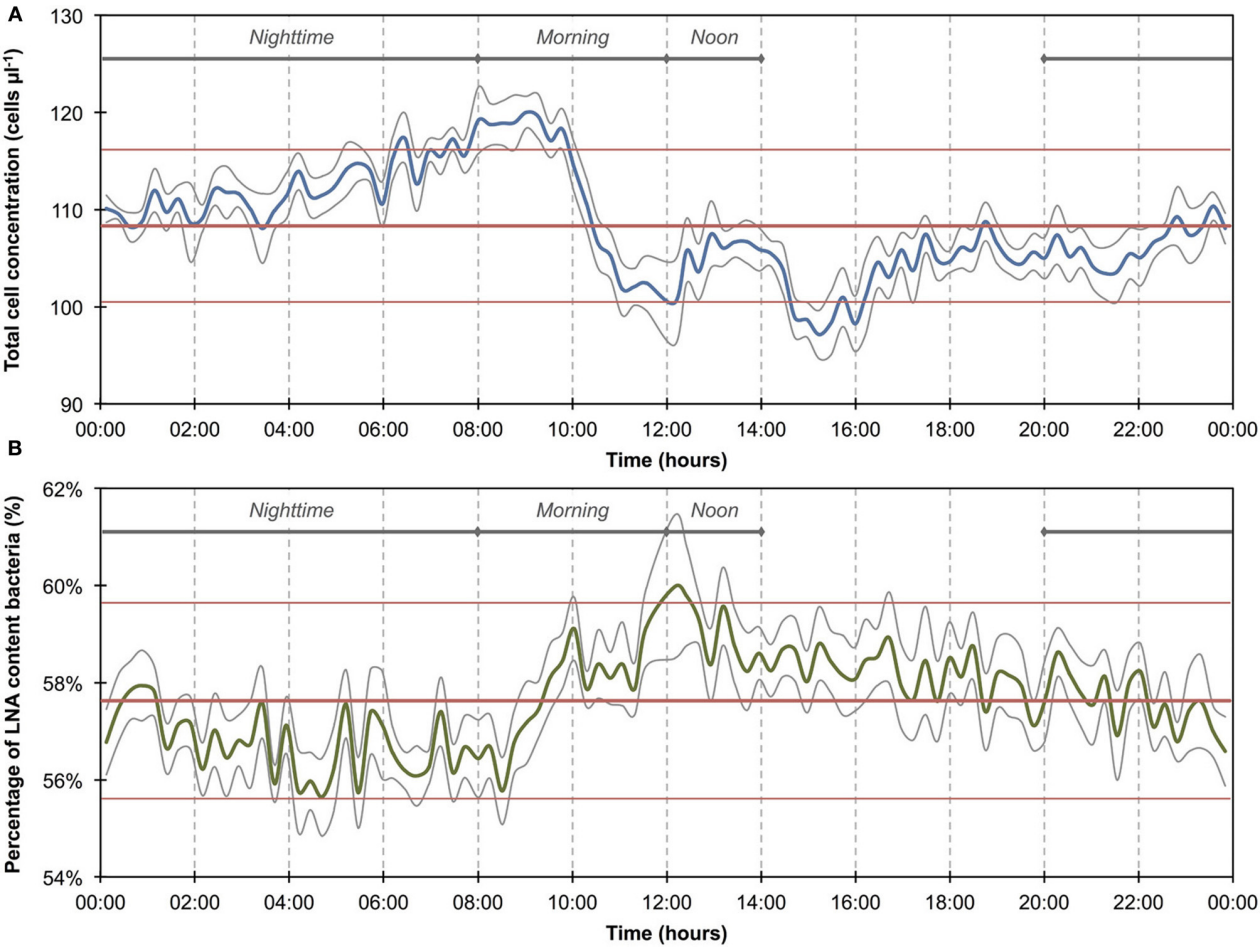
**Baseline data of the daily microbiological fluctuations in continuously flowing municipal tap water for total cell concentrations (A) and percentage LNA content bacteria (B)**. The data set (*n* = 674) comprises only weekday data, excluding weekends and the apparent event (Figure 1). Blue and green lines are 15-min averages of all included days for both parameters. Gray lines represent the standard error for each point of the average. Horizontal red lines show the average (thick line) and standard deviations (thin lines) calculated from all data combined (*n* = 674). A daily fluctuation is clearly detectable in both parameters.

The 1300 online FCM measurements of flowing municipal tap water during 14 consecutive days showed overall relatively stable TCC and fluorescence fingerprints [i.e., percentage of LNA content bacteria, Prest et al. (2013)], but also revealed daily fluctuations as well as a discernable event/shift in TCC between days 10 and 14 (Figure 1). In order to establish baseline data for this particular ecosystem, we extracted from the combined data set only the values recorded on weekdays, excluding the weekends and the apparent event (days 1–4, 7–10, 14–15; Figure 1 and Table S1). The resulting data set (*n* = 674) had an average TCC of 108.3 ± 7.8 cells μL^−1^ and an average percentage of LNA content bacteria of 57.6 ± 2.0%. These data were pooled and sorted in a 24-h window according to their individual measurement times. From this we calculated averages and standard error of the data points of all days within the same 15-min interval (Figure 2). This combined data revealed a clear daily pattern with respect to TCC (Figure 2A). During nighttime (20:00–08:00), the TCC gradually increased at a rate of approximately 0.8 cells μL^−1^ h^−1^, and values were mostly above the overall average. The morning hours (08:00–10:00) displayed the highest bacterial concentrations (119.0 ± 5.8 cells μL^−1^; *n* = 55), followed by a sharp decrease between 10:00 and 12:00, a noticeable peak around noon (12:00–14:00) and the lowest concentrations around 15:00. TCC tended to be below the average during daytime. The percentage of LNA content bacteria showed less daily fluctuation although a higher percentage of LNA content bacteria was observed during daytime compared to nighttime (Figure 2B). Only a weak negative PCC (−0.34, *n* = 674) was observed between the TCC and percentage of LNA. On day 10, a remarkable and sudden shift in TCC occurred, with average concentrations increasing to 145.1 ± 12.5 cells μL^−1^ (*n* = 410) and only gradually decreasing again until day 14. In contrast, the percentage of LNA content bacteria remained largely unaffected by the dramatic shift in cell concentration (Figure 1).

### Environmental ecosystem: river water

**Figure 3:**
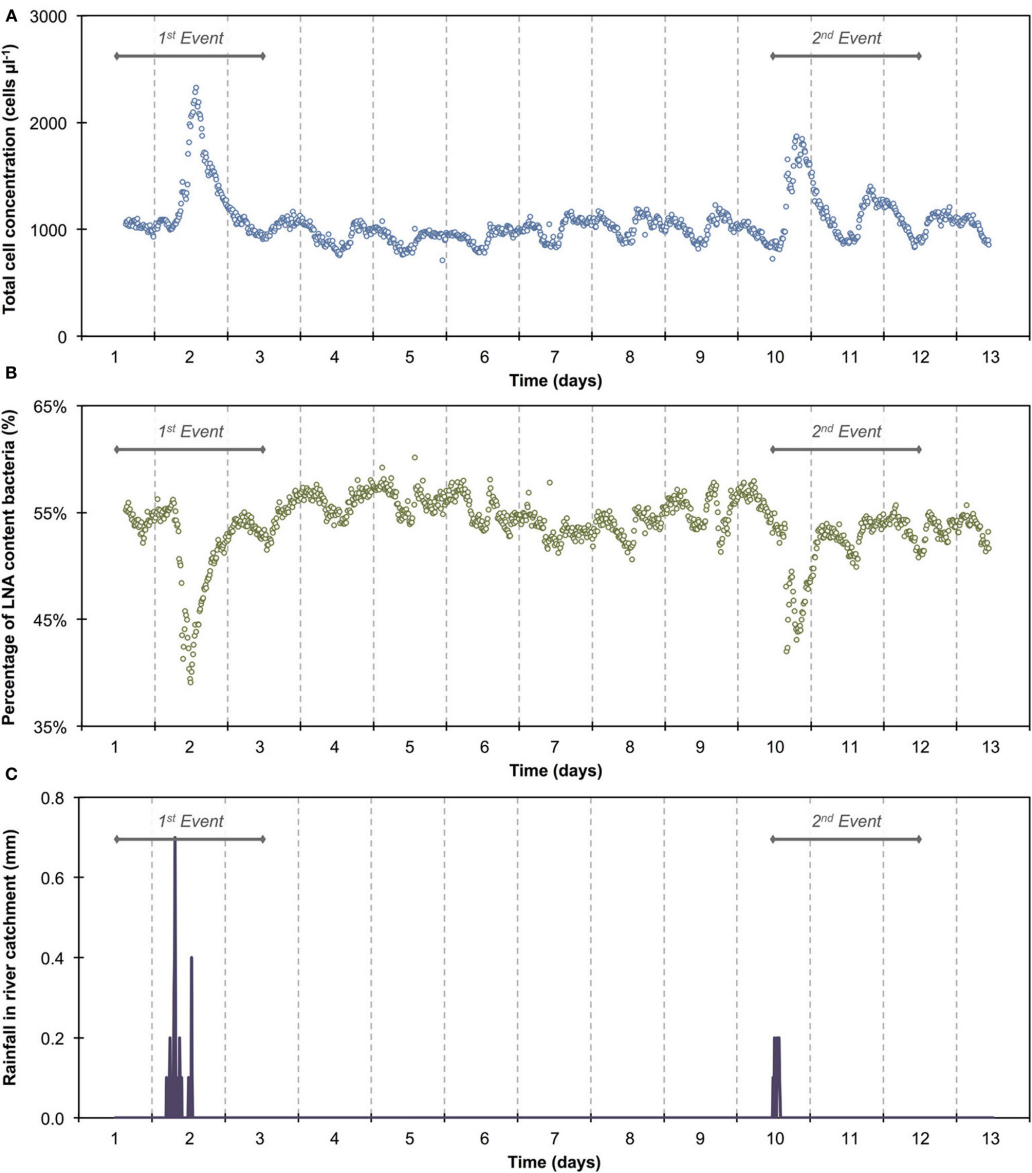
**Online FCM measurements of river water at 15-min intervals over 12 consecutive days, showing total cell concentrations (TCC) (A) and the percentage of LNA content bacteria (B) (*n* = 1104)**. Two distinct events can be seen on days 1–3 and 10–12, corresponding directly with two rainfall events in the river catchment **(C)** and resulting in an increase in TCC and decrease in the percentage of LNA content bacteria. In addition, repeated daily patterns are visible, which are much smaller in magnitude than the two events.

**Figure 4:**
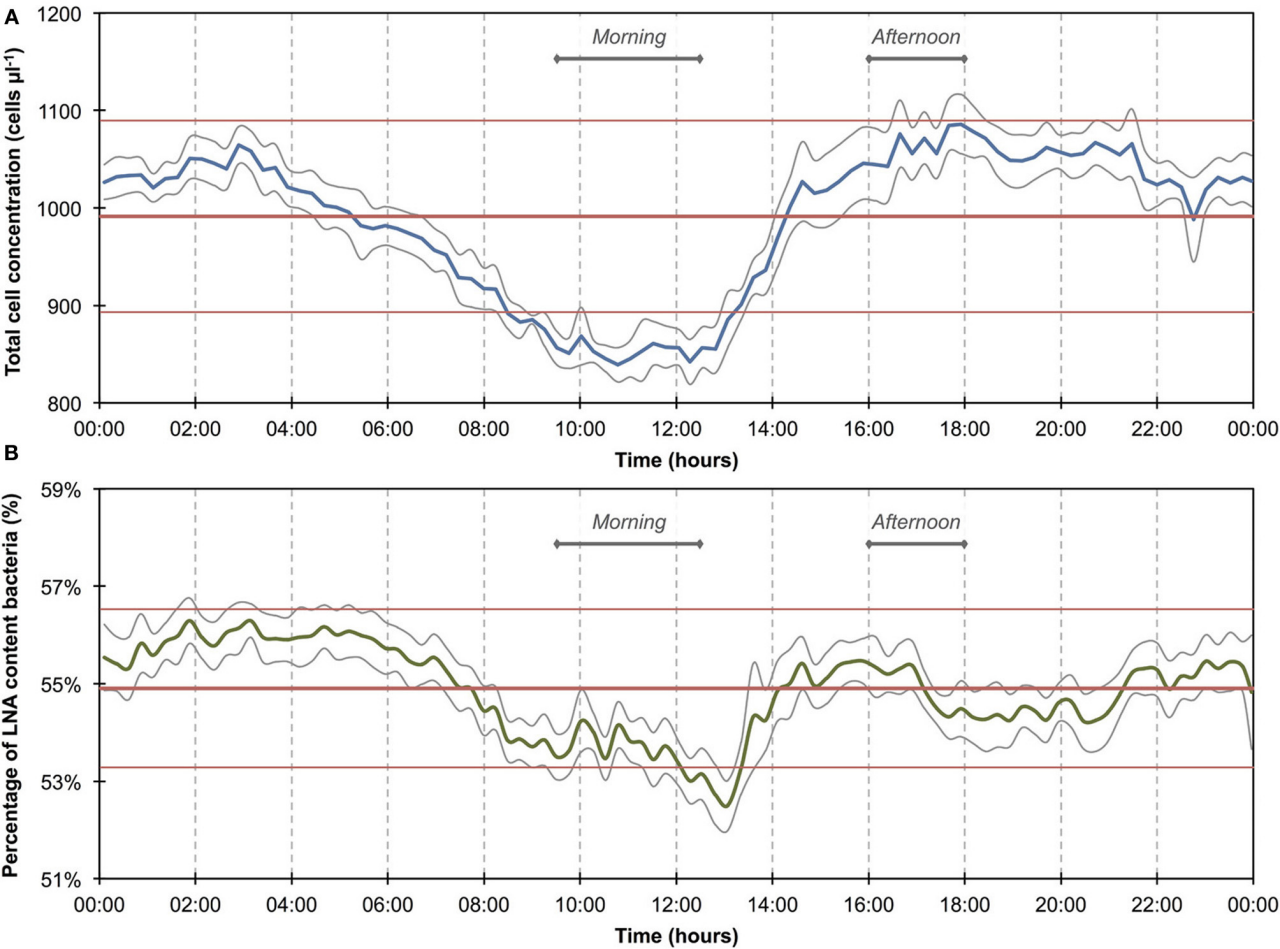
**Baseline data of daily microbiological fluctuations in river water for total cell concentrations (A) and percentage LNA content bacteria (B)**. The data set (*n* = 740) comprises only data outside of the two apparent events (Figure 3). Blue and green lines are 15-min averages of all included days for both parameters. Gray lines represent standard errors for each point of the average. Horizontal red lines show the average (thick line) and standard deviations (thin lines) calculated from all data combined (*n* = 740). A daily fluctuation is clearly detectable in both parameters.

The 1100 online FCM measurements of river water at 15-min intervals during 12 consecutive days showed overall stable TCC but revealed clear daily fluctuations and two very dramatic events (days 2 and 10). These were directly attributed to regional rainfall and were visible in considerable changes to both TCC and the percentage of LNA content bacteria (Figure 3). To assess this in detail, a baseline for this particular ecosystem was established by excluding the two rainfall events (days 1–3 and 10–12, Figure 3 and Table S1) and pooling the remaining data together as described above (Figure 4). For all baseline data combined (*n* = 740), the average TCC was 991.3 ± 98.2 cells μL^−1^, and the average percentage of LNA content bacteria was 54.9 ± 1.6%. Moreover, from the averaged data a clear pattern emerged, with the lowest TCC in the morning hours (09:30–12:30) at 854.1 ± 52.1 cells μL^−1^ (*n* = 84) and the highest concentrations in the afternoon (16:00–18:00) at 1063.1 ± 83.9 cells μL^−1^ (*n* = 63). During the dry periods (i.e., times without rainfall), the percentage of LNA content bacteria increased when the TCC increased (Figure 3), although the fluctuations were smaller than for the TCC. Relative to the baseline data, the two events (days 1–3 and 10–12; Table S1) were unmistakably clear. Both events were a direct consequence of acute bacterial loads following rainfall (Figure 3). Local weather station information showed a total of 3.1 mm of rain falling within 8 h on day 2 and a total of 1.4 mm of rain falling within 3 h on day 10 in the catchment area of the river (Figure S2). As a result, TCC increased above 2000 cells μL^−1^ on day 2 and above 1500 cells μL^−1^ on day 10. During the events, the trend in the percentage of LNA was dissimilar to the dry periods, and values dropped below 45% during both events as a direct consequence of increased concentrations of HNA content bacteria in the water. Values returned to the baseline within 24 h after the rainfall ceased. A set of raw FCM data from 48 h of measurements for the river water experiment is available in video format in the supplementary information (Section 9).

### Correlations with abiotic sensor data

**Figure 5:**
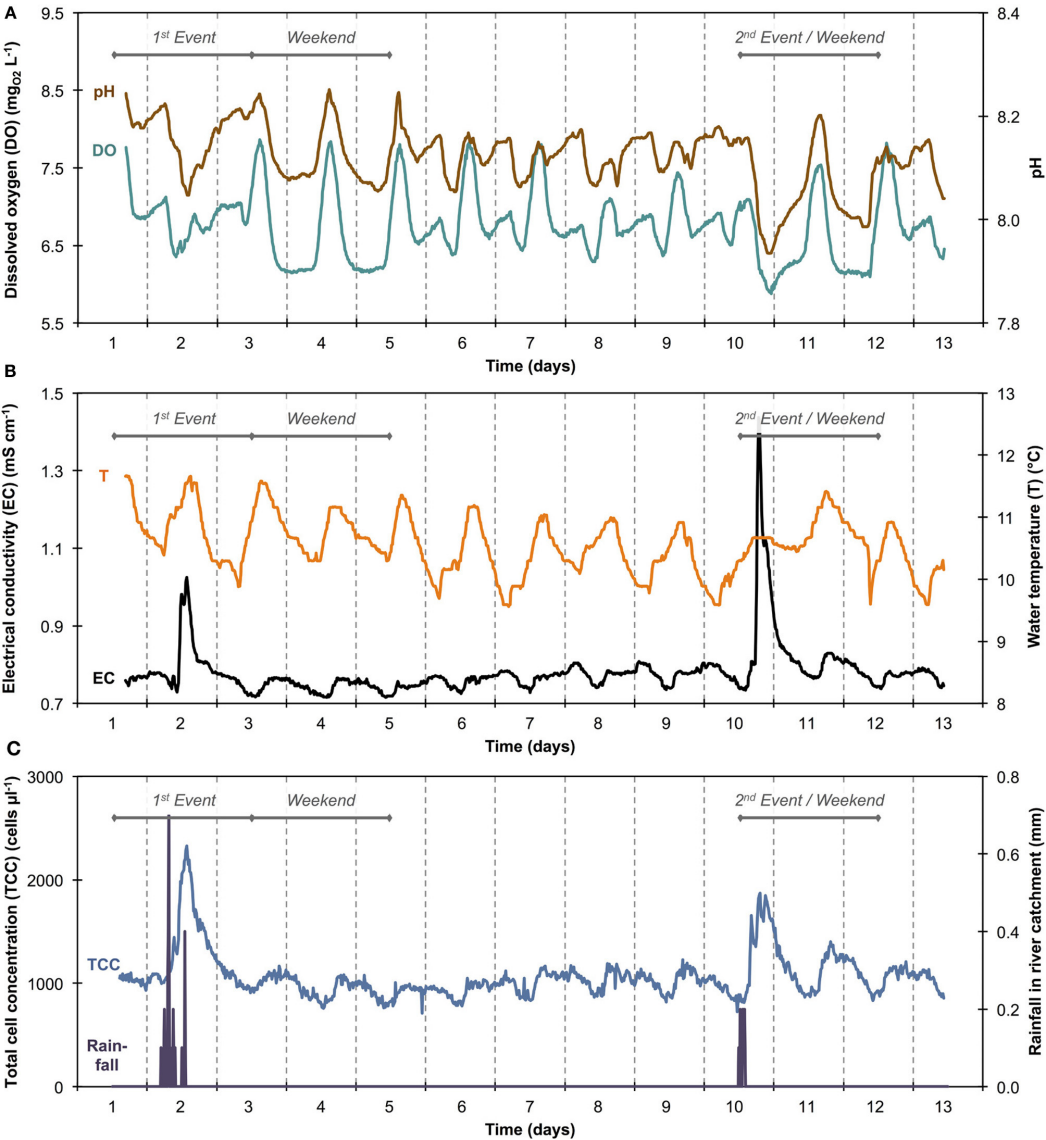
**Sensor data of river water for pH and dissolved oxygen (DO) (A) and electrical conductivity (EC) and temperature (T) (B)**. Total cell concentration (TCC) and rainfall data from Figure 3 are shown in simplified form **(C)** for easier and direct comparison with other sensor data.

**Figure 6:**
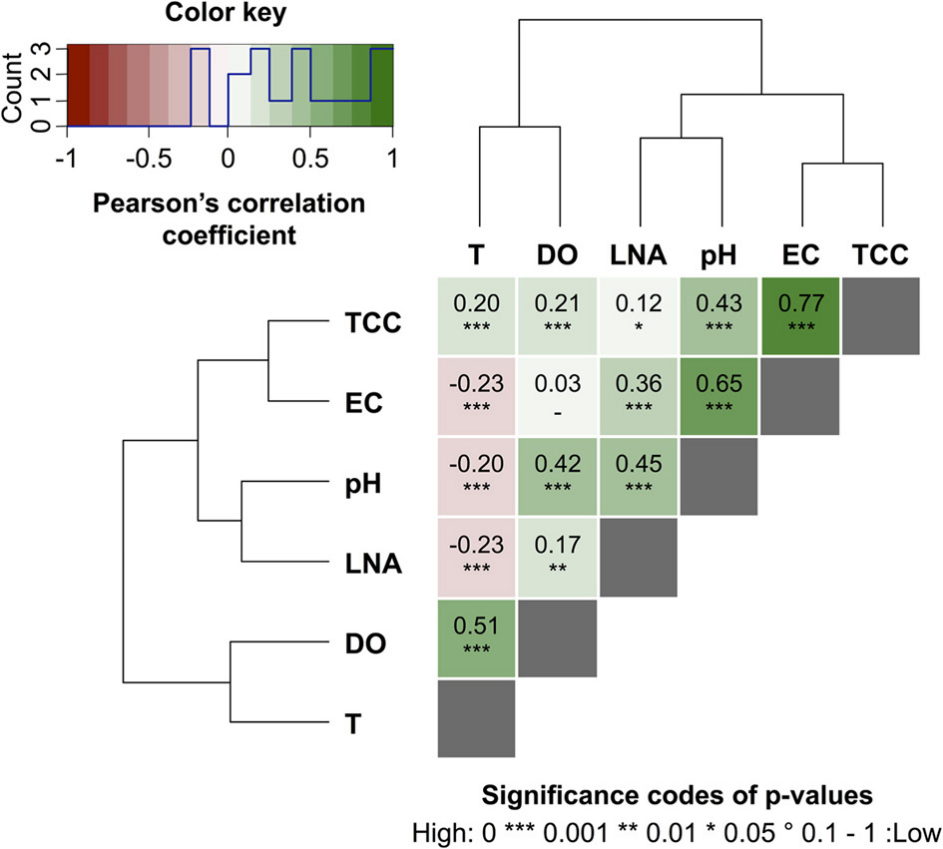
**Heat map of pair-wise Pearson's correlation coefficients (PCC) computed between online FCM measurements, i.e., total cell concentration (TCC) and percentage of LNA content bacteria LNA, and the abiotic online sensor parameters, i.e., temperature (T), dissolved oxygen (DO), electric conductivity (EC), and pH in river water (Chriesbach, Dübendorf, Switzerland)**. Hierarchical clustering using the Ward's algorithm was first applied to reorder all parameters in clusters according to their correlations as displayed by the dendrograms. The values and directions of the PCC are displayed according to the color key, i.e., positive correlations as green gradients from 0 to 1 and inverse correlations as red gradients from 0 to −1. Baseline data for these correlations were calculated based on 4 weekdays (days 6–9, Table S1).

Online data from four sensors for abiotic parameters complimented the online FCM data with respect to daily pattern analysis and event detection (Figure 5). Figures 5A,B show daily fluctuations in all abiotic parameters. Temperature, DO, and pH generally peak in the early afternoon and then keep dropping during the night until reaching a low point shortly before noon the following day. In contrast, EC showed a sharp drop in the morning with a low around noon, followed by a sharp increase in the afternoon and a steady increase overnight and into the next morning. The rainfall events were most obvious in the conductivity data (Figure 5B) with other parameters showing comparably little response. In fact, conductivity data correlated remarkably well with TCC data both between and during events (Figure S3). Some sensors also detected apparent weekend-related patterns (notably pH and DO) that were not seen in the FCM data (Figure 5). This was ascribed to wastewater treatment discharge upstream of the sampling point. Hence, for a simplified direct multivariate comparison of all biotic and abiotic data with respect to the daily patterns, we selected only a 4-day period (days 6–9), excluding weekends and rainfall events (Figure 3 and Table S1). The resulting data set (*n* = 373) displayed some interesting relationships between biotic and abiotic parameters (Figure 6). Since the two statistical approaches, i.e., Pearson's linear correlation and Spearman's rank-order correlation, were providing similar trends, linear relationships between parameters were assumed. Firstly, as suggested above (Figure S3), EC correlated positively with TCC (PCC = 0.77). Secondly, pH and the percentage LNA content bacteria showed a weaker but significant correlation (PCC = 0.45). Thirdly, the dendrogram clustering suggests that EC and pH were affiliated with TCC and the percentage of LNA content bacteria. Based on the calculated *p*-values computed at 95% confidence level, the majority of the determined correlations were highly significant. Only weak correlations with low significance were found between the biotic parameters and T and DO. More detailed results for the different parameters are also shown in Figure S4.

## Discussion

Two distinctively different aquatic ecosystems were automatically sampled and measured during multiple days involving minimum human intervention and resulted in FCM data sets in excess of 1000 data points for each experiment. This allowed the establishment of baseline data and the subsequent detection of cyclic patterns, gradual changes, and sudden events for each ecosystem. The automated approach described herein extends the state-of-the-art of *in-situ* monitoring of microbial dynamics in complex engineered and environmental ecosystems considerably. It furthermore allows for a meaningful combination of microbial data with widely applied online sensor data of abiotic parameters.

### Bacterial dynamics in drinking water

The average TCC measured in this study corresponded to typical values for non-chlorinated drinking water (Hammes et al., 2008; Lautenschlager et al., 2010; Vital et al., 2012). We observed a reproducible daily pattern in the data (Figures 1, 2) as well as an unexplained dramatic event in the second week of measurements. To our knowledge, no previous studies investigated tap water at such high temporal resolution, and therefore the interpretation of these dynamics is somewhat speculative. It was shown before that overnight stagnation in buildings leads to an increase in TCC and in the percentage of HNA content bacteria (hence by default a decrease in the percentage of LNA content bacteria), and also that flushing of taps leads to a decrease in both these parameters (Lautenschlager et al., 2010; Prest et al., 2013). This mirrors the general TCC and percentage of LNA content bacteria behavior seen in Figure 2. While the tap measured in the present study was continuously flowing, the remainder of the building was indeed subject to overnight stagnation. In fact, there is additional evidence that the FCM patterns followed localized water usage to some degree: (1) a dramatic decrease in TCC during the morning hours when most people normally arrive at work; (2) a peak during lunchtime which would correspond to a change in water usage; (3) the fact that weekends showed clearly dissimilar patterns to weekdays (Figure S5). All these fluctuations could relate to a combination of regrowth and changes in bacterial attachment/detachment due to changing hydraulic conditions on local or regional level (Lehtola et al., 2006). The dramatic increase in TCC on day 10 was unexpected. Discussions with the local building managers as well as the regional water authority yielded no additional information on any specific event during this period. The high frequency FCM data revealed previously unknown dynamics in the drinking water and suggests the investigation of detailed cause-effect relationships in future studies.

### Bacterial dynamics in river water

The average TCC measured in this study corresponded to many previous measurements of this particular river and is in the normal range of surface water (Wang et al., 2007). The data show an apparent daily fluctuation (Figures 3, 4) as well as two dramatic events linked to rainfall. Sensors for abiotic parameters also detected daily fluctuations and to some extent the rainfall events (Figure 5). The occurrence of diurnal fluctuations in the abiotic parameters (DO, EC, and pH) has been described in detail for rivers, and is attributed to microbial photosynthetic and respiratory activities coupled to radiation and to subsequent changes in ion concentrations due to changes in precipitation and dissolution of calcite [for details see Vogt et al. (2010) and Hayashi et al. (2012)]. While we are not aware of any previous studies on fluctuations in the TCC at a similar time resolution, many of the processes that drive the abiotic fluctuations are likely to affect the microbiology as well. It is generally recognized that carbon compounds released by algae into the water during their daytime activity serve as nutrients for bacteria and thus induce growth (Sundh and Bell, 1992). Algal mats and biofilms are present in this shallow river, which are likely to produce substantial amounts of carbon compounds. In addition, higher temperatures during the day may also favor increased bacterial growth. The measured increase in planktonic bacterial concentration is most likely due to increased detachment from river biofilms that could show increased growth for the reasons stated above. Figure S3A shows a clear positive correlation between conductivity and bacterial cell concentrations in dry periods and during rainfall events. Such correlations have been described in literature (Krishnamurti and Kate, 1951), although it is not clear whether the observed correlation in the present study was due to direct or indirect influences. Moreover, correlations during rainfall events are likely to have different causes from correlations during normal periods. During rainfall events, run-off from the surrounding catchment (including urban sewers) flows into the river and transports additional ions but also bacteria into the water body. This can explain the changes observed in the HNA/LNA pattern observed in Figure 3. However, during longer rainfall events, a dilution effect of relatively “pure” rainwater may occur after the initial peak due to run-off.

### Considerations on instrument artifacts

Although the aim of the present study was not explicitly to assess cause-effect relationships in the studied ecosystems, one logic question is whether the cyclic patterns that we observed were actual events, or unexplained instrumental artifacts of the FCM or staining robot. We believe that the latter was not the case. Firstly, the changes in TCC in the river water do not follow the same pattern as the changes in the tap water (Figures 2, 4, Figure S6). Secondly, the relationship between the percentage of LNA content bacteria and TCC are different in the two ecosystems. Whereas in the tap water the percentage of LNA content bacteria tended to decrease with increasing TCC, the river water showed an opposite tendency (Figures 3, 4). Thirdly, the weekend did not show the same behavior in the tap water compared to weekdays (Figure S5). Finally, the sensors for abiotic parameters measuring the river water showed cyclic events as well (Figure 5 and Figure S4, discussed above). In addition, we did a comparison of automated FCM measurements with conventional manual FCM measurements, which showed similar data measured with both approaches (Figure S7). All these points strongly suggest that the changes/patterns were real and inherent to the ecosystems that were investigated.

### Advantages of automated online FCM measurements

The experiments demonstrated several advantages of online FCM in monitoring:

- *Long-term measurements* allowed for the detection of stable phases, cyclic patterns and unexpected events, which would either be missed or incorrectly characterized by occasional grab sampling (Figures 1, 3). Events are not necessarily predictable in time and magnitude. It is thus ideal to use such long-term data to establish baselines that enable the identification and characterization of events as deviations from that baseline. As was shown in the basic data processing above, this can give a clear indication of critical time periods to be sampled/analyzed in more detail.
- *Frequent measurements* allow for detailed analysis of events and fluctuations (Figures 2, 4). This is, for example, crucial when analyzing a flood event and comparing width and height of peaking curves of different parameters (Stadler et al., 2008; Butscher et al., 2011) or the relationship between different microbial parameters (e.g., TCC and the fluorescence fingerprint as highlighted for both experiments above).
- *Automated measurements* allow for the recording of expected or unexpected events (e.g., snowmelt, rainfall) independent of the availability of personnel on location (Figure 3). Major events of interest may be rare or untimely and thus reliable automated systems increase the chance of measuring such events, even in remote locations (Stadler et al., 2008). In addition, automated systems free up human resources for other (non-automated) measurements in parallel during an event (e.g., conventional plating, ATP-measurements).
- *Online measurements* allow real-time, *in-situ* monitoring of a variety of aquatic ecosystems that display temporal variability at different time scales, concentration ranges, and microbial community compositions. With both daily variations and specific events, two types of dynamics could be monitored in this study. Measurements were in the range of 10^5^ and 10^6^ cell mL^−1^ respectively and based on an earlier evaluation of FCM can be assumed to be accurate and sensitive (Hammes et al., 2012). The percentage of LNA content bacteria, as one simplistic form of FCM fingerprinting (SLMB, 2012; Prest et al., 2013), differed clearly between the two investigated ecosystems and within cycles and events. More advanced analysis of differences between systems and in time can be applied for example through extended fingerprinting methods (Koch et al., 2013a).
- *Multi-parameter measurements*, i.e., online FCM combined with other online sensors (e.g., temperature, conductivity), allow for robust differentiation between aquatic ecosystems and/or their respective conditions as well as identification of stability and deviations from it (Figures 2, 4). The combined biotic and abiotic data sets for the river water show interesting correlations and offer first insights into cause-and-effect relationships within the ecosystem (Section Correlations with Abiotic Sensor Data and Bacterial Dynamics in River Water, Figures 5, 6, and Figure S3).

### Challenges with respect to implementation and method establishment

FCM analysis of aquatic microorganisms has evolved considerably during the last decade (Hammes and Egli, 2010). However, despite the described advantages and the proof-of-concept data shown here (Figures 1, 3) and elsewhere (Hammes et al., 2012; Brognaux et al., 2013), routine application of online FCM is not yet reality. Several challenges with respect to implementation remain, and these differ for applications as a laboratory research tool and for application as a routine monitoring/sensor tool for water utilities.

Firstly, conventional FCM hardware is still relatively expensive (20–50 kUSD) today, although it is expected to decrease in price in the future. For the system described herein, we estimated operating costs of approximately 0.1 USD per measurement (thus < 10.0 USD per day), excluding instrument purchase (Table S8). The automated staining robot used herein was a prototype that is not currently commercially available, but construction of similar systems is clearly feasible (Hammes et al., 2012; Brognaux et al., 2013).

Secondly, the applicability of the automated system to different aquatic ecosystems is limited with respect to cell concentrations and background levels in the samples of interest. Commercially available bench top flow cytometers typically measure in a range of 10^3^–10^6^ cells mL^−1^ (Hammes et al., 2008, 2012), which is suitable for a variety of aquatic ecosystems such as groundwater, tap water, and rivers and lakes (Wang et al., 2007). However, for more concentrated samples [e.g., in bioreactors or activated sludge settings (Koch et al., 2013b)], extensive dilution techniques would need to be included (Abu-Absi et al., 2003; Broger et al., 2011). This potentially adds costs and measurement error to the system. In general, none-bacterial particles may cause clogging of instruments and overlaying of background and bacterial signals (Hammes and Egli, 2010). Although we experienced no problems in the current study, this may be of particular importance in event monitoring, where for example rain events may cause substantial turbidity in the sampled water (Pronk et al., 2006).

Thirdly, monitoring with automated online FCM generates data sets comprising hundreds-to-thousands of data points for several parameters (e.g., TCC, green/red fluorescence intensity, forward/sideward scatter). It has previously been shown that standardization throughout the FCM analysis train (including data processing) is essential for high quality data (SLMB, 2012; Prest et al., 2013). Efficient, standardized data processing and analysis will be crucial to make optimal use of the newly available richness in microbial data. Our data sets were already so large that we required custom software for processing and extension of this can include advanced fingerprinting methods (Koch et al., 2013a; Prest et al., 2013) and time series analysis (Pomati et al., 2013). Through the quantification of microbial variability (e.g., temporal, geographical), relationships between observed patterns and potential drivers (e.g., biofilm attachment and detachment, contamination, mixing, treatment methods, temperature) can be tested systematically.

### Applications

We envisage a broad range of future applications of fully automated online FCM. Firstly, in treatment process optimization but also routine operation of drinking water/wastewater treatment plants, online microbial monitoring can help to investigate different modes of operation or the influence of diurnal process changes or changing raw water quality (Hammes et al., 2008; Vital et al., 2012). Similar applications can be of value in related industries such as bottled water production, beer brewing or the production of ultra-pure water. Secondly, in environmental aquatic ecosystems, interactions between abiotic and biotic factors both for regular and event-based dynamics can be studied with considerable detail using this approach (Pronk et al., 2006; Stadler et al., 2010; Butscher et al., 2011). Thirdly, automated measuring of microbial parameters renders event monitoring for quantitative microbial risk assessment considerably much more feasible (Signor and Ashbolt, 2006). Finally, a large variety of laboratory-based research can benefit from high temporal resolution and automation. Straightforward examples are experiments on bacterial batch growth or continuous cultures under different conditions (e.g., different media, inhibiting agents) or experiments looking at different types and intensities of disinfection (Arnoldini et al., 2013).

## Conclusions

Our results demonstrate that automated *in-situ* FCM analysis is feasible for the investigation of dynamic aquatic ecosystems at high temporal resolution during multiple days.

The resulting large sets of reliable, quantitative microbiological data considerably improved characterization of dynamic fluctuations, which in turn enhances our understanding of complex engineered and environmental ecosystems.

The data revealed both daily fluctuations and specific events in the drinking water and river water ecosystems, suggesting that temporal variations should be carefully taken into consideration during sampling and analysis of aquatic ecosystems.

Automated FCM allows for the combination of microbial data at high temporal resolution with conventional online parameters, thus expanding the horizon toward a complete sensing approach.

## Author contributions

Hardware/software development: Bradley E. Kratochvil, Mathias S. Weyland. Experimental design: Michael D. Besmer, Jürg A. Sigrist, Frederik Hammes. Research: Michael D. Besmer, David G. Weissbrodt, Jürg A. Sigrist, Frederik Hammes. Data analysis: Michael D. Besmer, David G. Weissbrodt, Mathias S. Weyland, Frederik Hammes. Writing/editing: Michael D. Besmer, David G. Weissbrodt, Bradley E. Kratochvil, Jürg A. Sigrist, Mathias S. Weyland, Frederik Hammes.

### Conflict of interest statement

The authors declare that the research was conducted in the absence of any commercial or financial relationships that could be construed as a potential conflict of interest.
