# Supplementary material for: The feasibility of automated online flow cytometry for in-situ monitoring of microbial dynamics in aquatic ecosystems
Source: Front Microbiol. 2014 Jun 2;5:265. doi: 10.3389/fmicb.2014.00265 (PMC4040452; doi:10.3389/fmicb.2014.00265)
Supplement: Supplementary file 1 [file DataSheet1.PDF]

Supplementary information:

The feasibility of automated online flow cytometry for *in-situ* monitoring of microbial dynamics in aquatic ecosystems

Michael Domenic Besmer, David Gregory Weissbrodt, Bradley Eugene Kratochvil, Jürg Andreas Sigrist, Mathias Sebastian Weyland, Frederik Hammes

Contents:

1. Definition of relevant time periods for the tap and river water datasets
2. Catchment area of the investigated river
3. Correlations between electrical conductivity and total cell concentrations
4. Baseline data for microbial and abiotic data in river water
5. Differences between weekdays and weekends in the drinking water dataset
6. Further considerations on instrument artifacts
7. Cost estimations of automated online FCM
8. Details on data extraction and analysis
9. Movie of density plots from river water experiment

1. Definition of relevant time periods for the tap and river water datasets

Table S1: Definition of relevant time periods for the tap and river water datasets respectively. From left to right the columns state the name of the period, the parameters that are displayed for the period, the exact definition of the period, the number of data points within the period, and the figures in which the period appears.

Tap water

Time period	Parameters	Days (hours) / hours	n=	Figures
All data	TCC, %LNA	1 (12:00) - 15 (12:00)	1'302	Figure 1
Event	TCC	10 (06:00) - 14 (16:00)	410	Figure 1
Weekends	TCC, %LNA	5 (00:00) - 7 (08:00), 12 (00:00) - 14 (08:00)	436	Figure 1
1st week-end	TCC, %LNA	5 (00:00) - 7 (08:00)	218	Figures 1 & S5
Baseline none-event weekdays	TCC, %LNA	1 (12:00) - 4 (24:00), 7 (08:00) - 10 (06:00), 14 (16:00) - 15 (12:00)	674	Figures 2, S5, S6
Nighttime	TCC	20:00 - 08:00	338	Figure 2
Morning	TCC	08:00 - 10:00	55	Figure 2
Noon	TCC	12:00 - 14:00	55	Figure 2

River water

Time period	Parameters	Days / hours	n=	Figures / tables
All data	All parame-ters	1 (14:15) - 13 (10:45)	1'104	Figures 3 & 5
Events	TCC, %LNA, EC	1 (12:00) - 3 (12:00), 10 (12:00) - 12 (12:00)	364	Figures 3, 5, S3
Weekends	All parame-ters	3 (12:00) - 5 (12:00), 10 (12:00) - 12 (12:00)	372	Figures 5 & S5
Baseline none-event days	TCC, %LNA, EC	3 (12:00) - 10 (12:00), 12 (12:00) - 13 (10:45)	740	Figures 4, 6, S3, S6
Baseline none-event weekdays	All parame-ters	6 (00:00) - 9 (24:00)	373	Figures 7, S4, S5
Morning	TCC	09:30 - 12:30	84	Figure 4
Afternoon	TCC	16:00 - 18:00	63	Figure 4

2. Catchment area of the investigated river

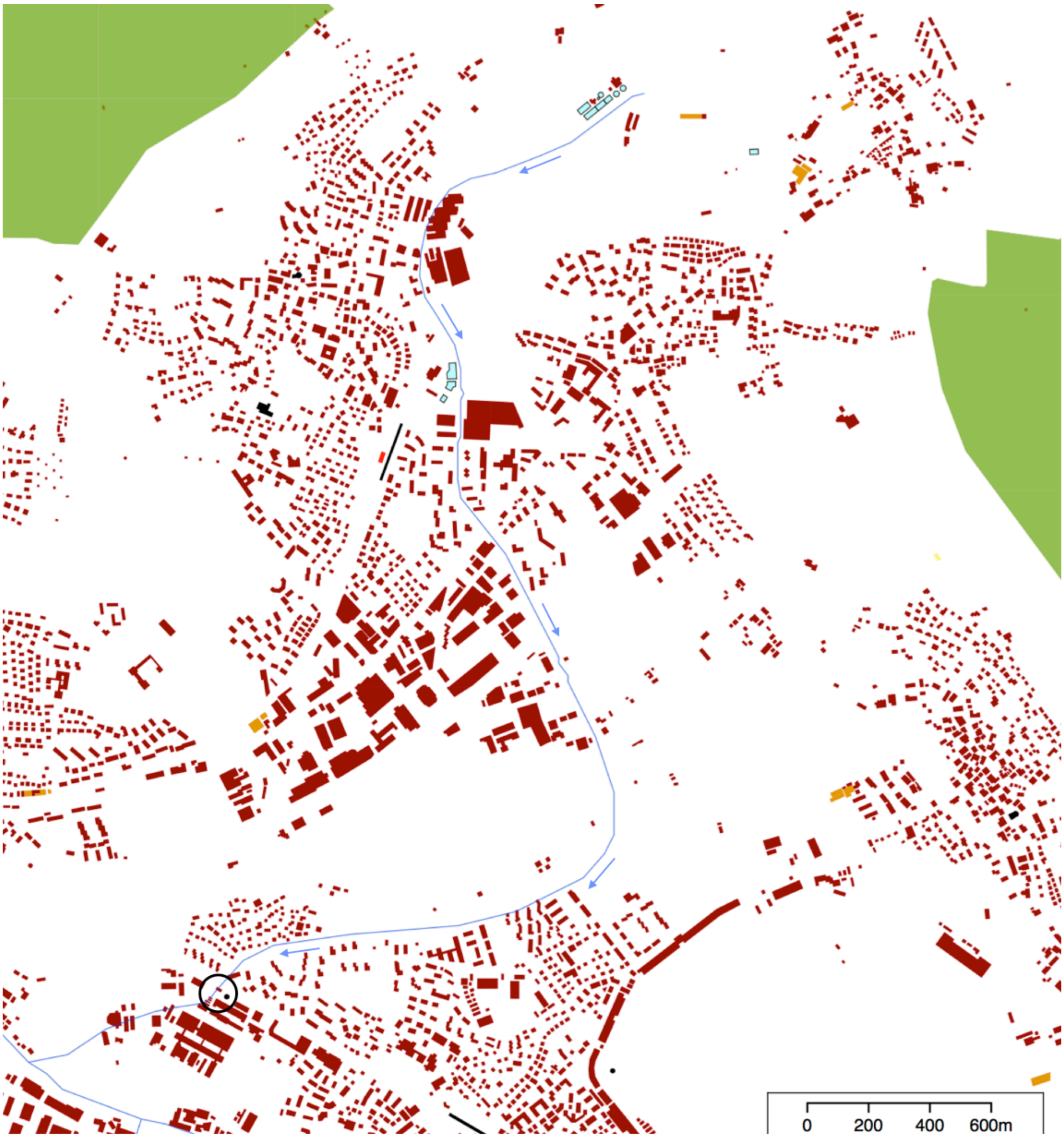

Figure S2: Catchment area of the investigated river (Chriesbach, Dübendorf, Switzerland). The river is indicated in blue and flows from north to south and later turns towards the west (blue arrows) where it discharges into a larger river. Buildings are indicated as red bodies and forest areas are displayed in green (east and north-west). The sampling point is indicated by a black circle in the lower part of the river (south-west). Source: geodata © swisstopo

3. Correlations between electrical conductivity and total cell concentrations

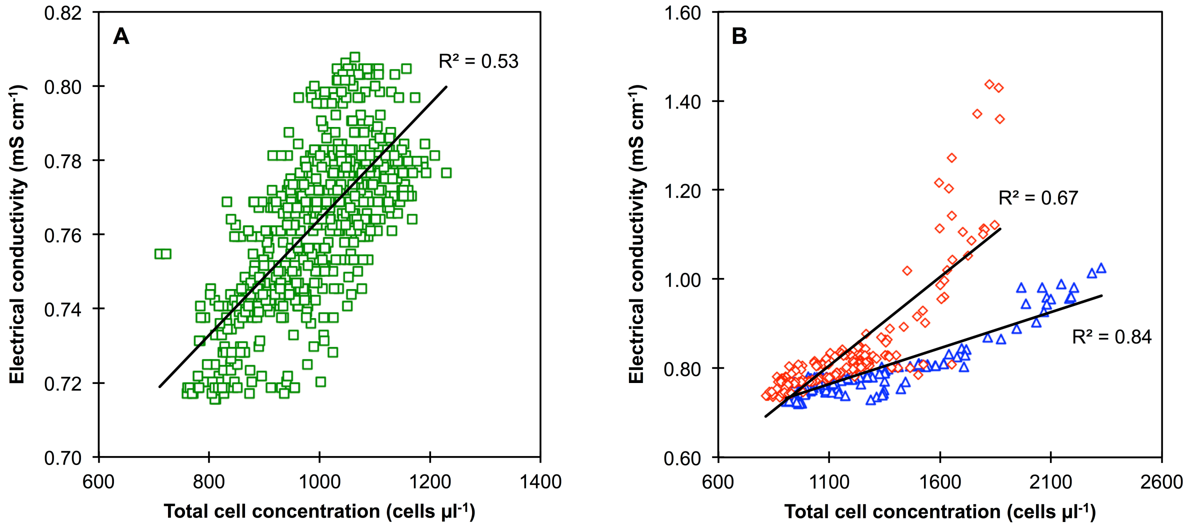

Figure S3: Linear relationships between electrical conductivity (EC) and total cell concentration (TCC) for non-event days (**A**) and the two rainfall events (**B**) in river water (Chriesbach, Dübendorf, Switzerland). Red diamonds represent the second event and blue triangles represent the first event (see Figure 5 and Table S1).

4. Baseline data for microbial and abiotic data in river water

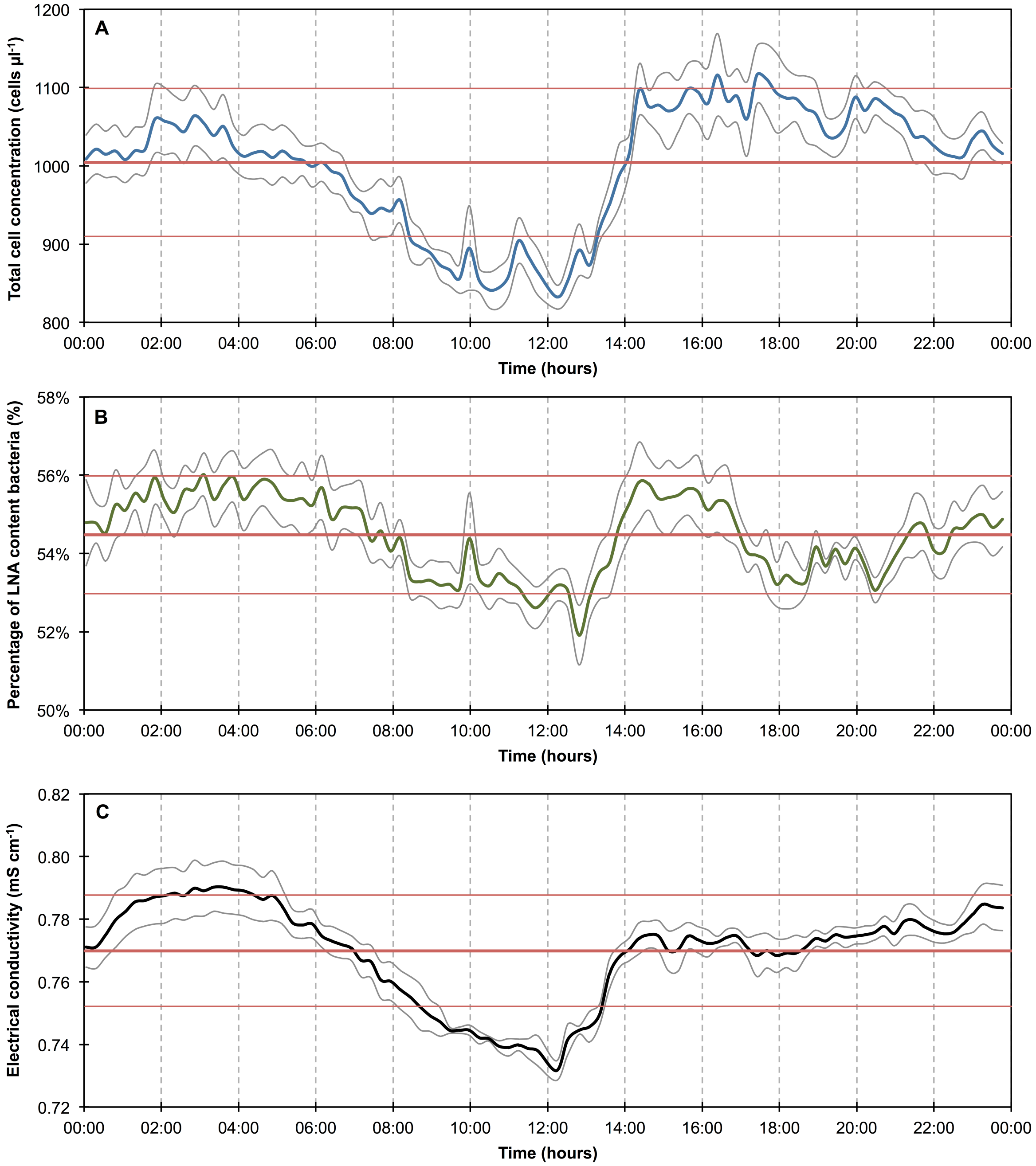

Figure S4: Baseline data of river water (Chriesbach, Dübendorf, Switzerland) for total cell concentration (**A**), percentage low nucleic acid (LNA) bacteria content bacteria (**B**), electric conductivity (**C**), temperature (**D**), pH (**E**), and dissolved oxygen (**F**).

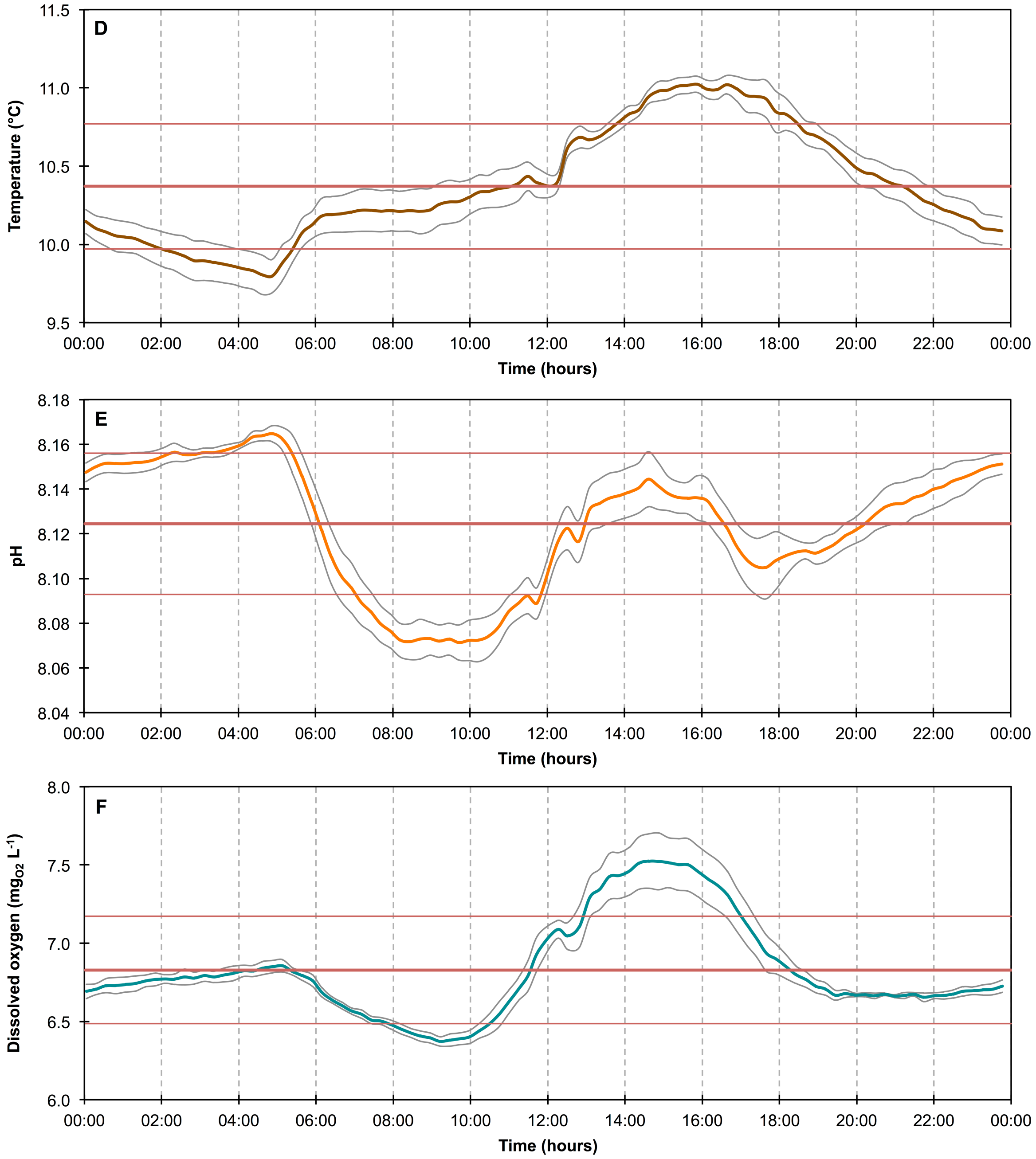

Figure S4: Baseline data of river water (Chriesbach, Dübendorf, Switzerland) (continued).

5. Differences between weekdays and weekends in the drinking water dataset

The trends in total cell concentration (TCC) on weekends compared to weekdays suggest that local water usage influenced the TCC. The tap water was sampled in a research building that is predominantly occupied during normal working hours and working days. Figure S5 shows significantly ($p < 0.0001$) higher average TCC on weekends (118.6 ± 6.2 cells μL^{-1}; n = 218) when considerably less water is used in the building and more stagnation is expected throughout the building compared to weekdays (108.3 ± 7.8 cells μL^{-1}; n = 674). While the trend is clear, more weekend measurements would ideally be required to confirm these patterns and the exact causes thereof.

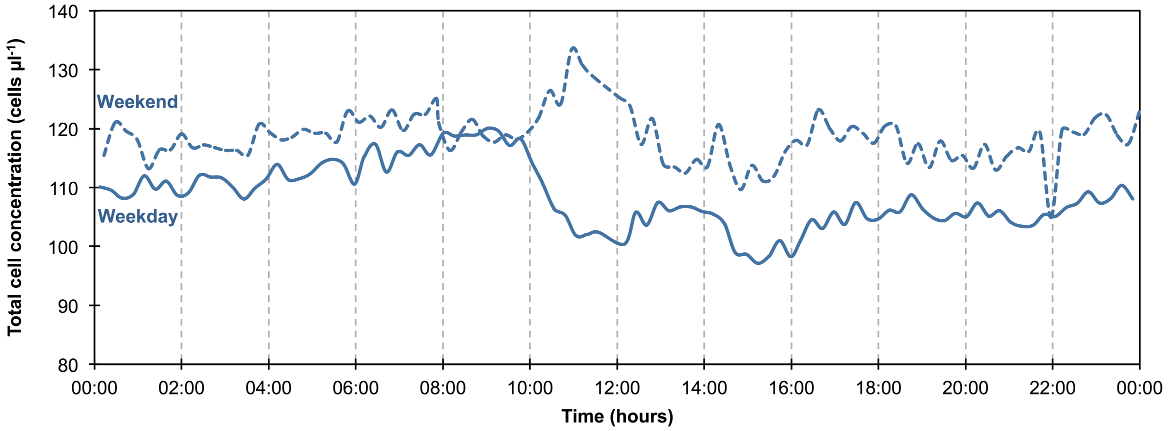

Figure S5: Comparison of the total cell concentration changes in tap water on weekdays (solid line) and weekends (dashed line). Data points for weekdays are the 15-min averages of all included days taken from Figure 2 (in total derived from 674 data points). Data points for weekends are 15-min averages of all included days from 24:00 on Friday to 08:00 on Monday (in total 218 data points).

6. Further considerations on instrument artifacts

If the observed daily patterns were instrumental artifacts, one would expect that the relative changes in the measured data would follow similar patterns, which is not the case. Figure S6A shows total cell concentration changes occurring at clearly different times and different rates during the day in the respective ecosystems, while Figure S6B shows completely dissimilar behavior in the percentage of low nucleic acid content bacteria between the river water and tap water measurements.

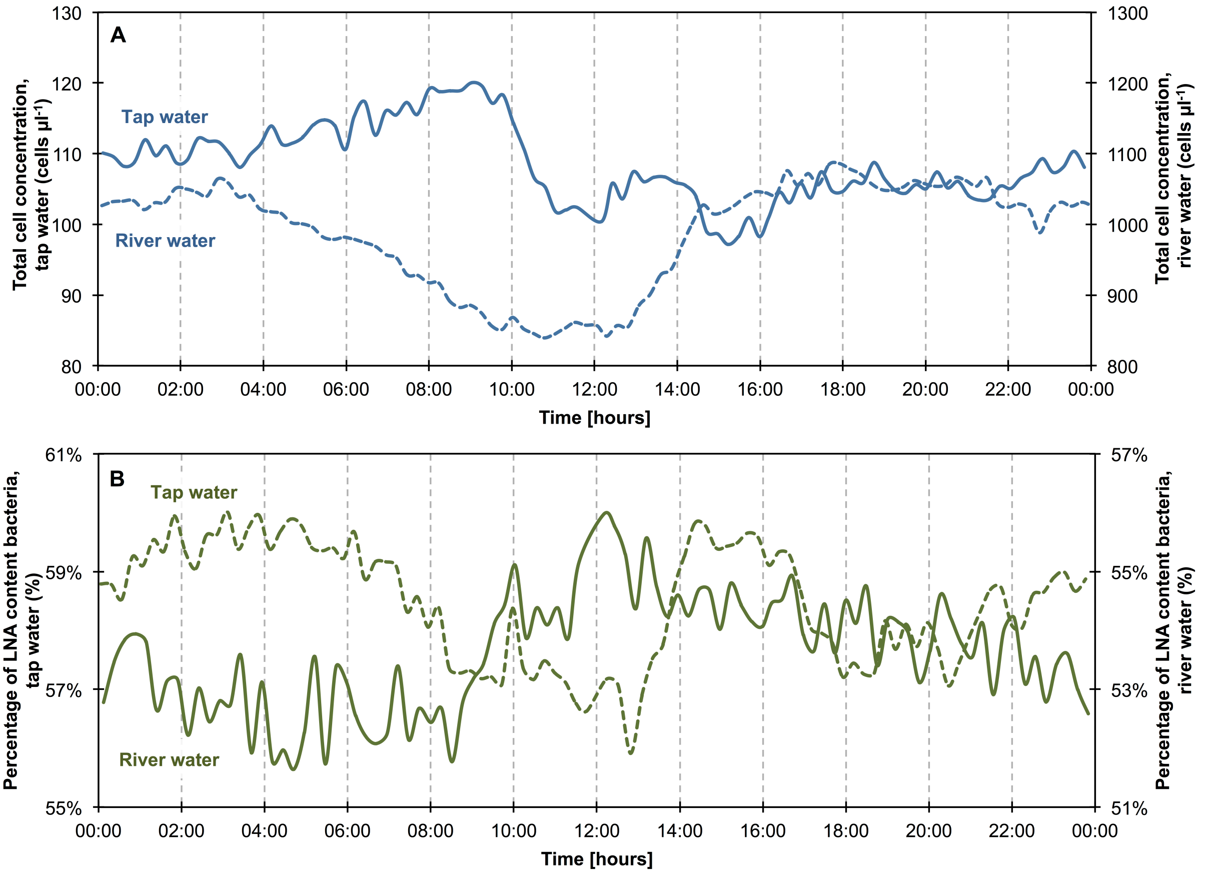

Figure S6: Comparison of the relative changes in the total cell concentrations (**A**) and the percentage of low nucleic acid (LNA) content bacteria (**B**) in the river water (dashed lines) and tap water (solid lines). Data points are 15-min averages of all included days taken from Figure 2 and Figure 4 in the main manuscript.

Direct comparison measurements were carried out to further exclude instrument artifacts caused by automation. In short, the automation device was connected to a bottle of drinking water from which it measured 10 samples in 15-min intervals. In parallel, a sample was collected from the bottle by hand and measured on a second Accuri C6 flow cytometer. The same was then repeated with a bottle of river water.

As can be seen in Figure S7, the automated measurements do not deviate systematically from the manual measurements. The consistently higher numbers for both TCC and percentage of LNA content bacteria for the automated measurements is due to a confirmed, slight difference in instrument calibration and has nothing to do with the automation.

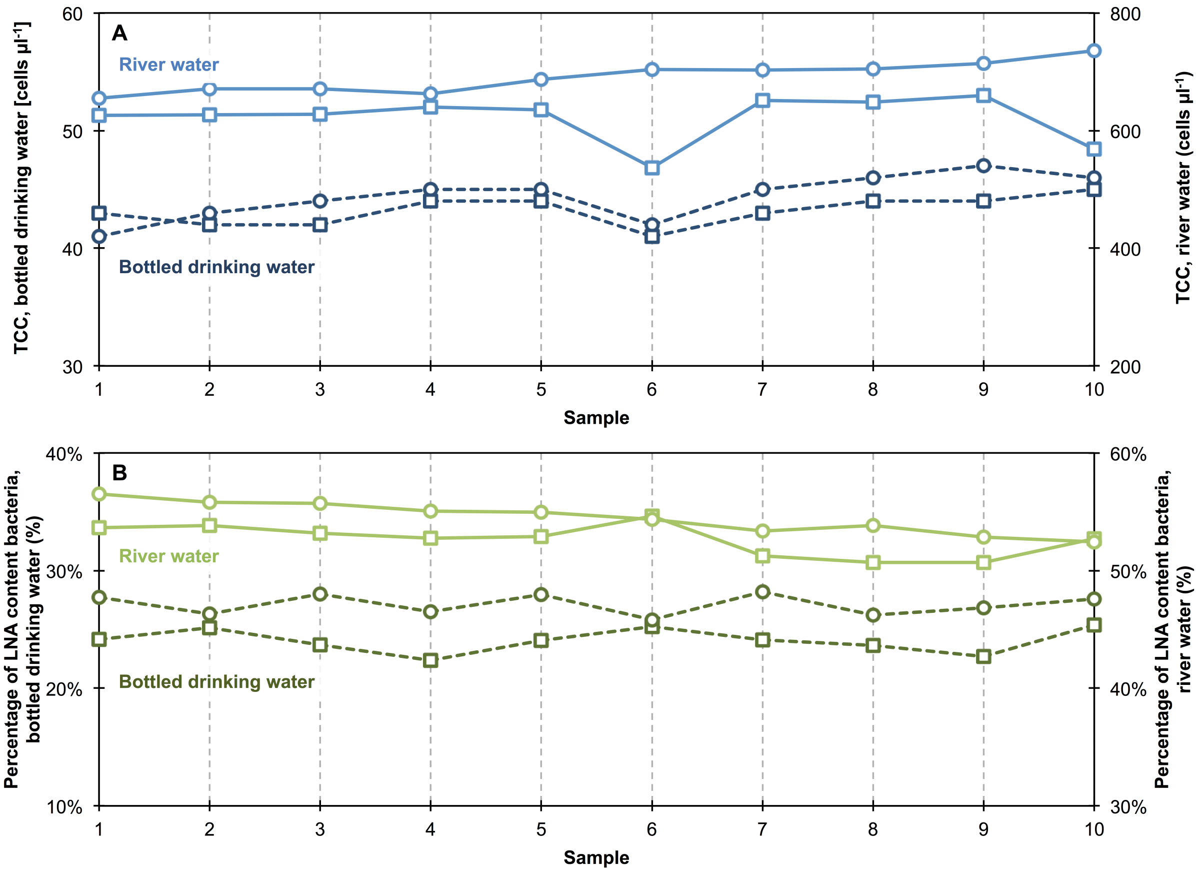

Figure S7: Comparison of the automated (squares) and manual (circles) FCM measurements for total cell concentrations (TCC) (**A**) and the percentage of low nucleic acid (LNA) content bacteria (**B**) in river water (solid lines) and bottled drinking water (dashed lines) (n=10).

7. Cost estimations of automated online FCM

Table S8: Estimations of operation and maintenance costs of automated online FCM.

Entity	Cost for 100 measurements (USD)
Stain	3.50
TRIS buffer	0.35
Tubing	0.50
Biozide	3.00
Beads	0.10
Javel	0.05
Soap	0.50
Nanopure	0.30
Waste	0.30
**Total**	**8.60**

8. Details on data extraction and analysis

The FCM and sensor data sets were imported in R as csv file using the “read.csv2” function and were then subjected to standardization using the “decostand” function in order to transform the data set values relative to each other (i.e., scale into zero mean and unit variance with default margin of 2 according to the R-package). This transformation allows for the comparison between the standardized FCM and sensor data sets. The Pearson’s linear correlation coefficients (PCC) and Spearman's rank correlation coefficients (monotonic relationship) were thus computed in R between the standardized FCM and sensor datasets. Since in the present study the Pearson's linear approach and the Spearman's monotonic approach were providing similar trends, linear relationships between parameters were assumed.

The PCC provide the extent of linear correlation between two variables considered (e.g., TCC vs conductivity: 0.77). With the computation in R, ones can get in one table all the correlation coefficients calculated pair-wise between parameters (i.e., the heatmap matrix of correlation coefficient provided with values and colors in Figure 7 but not re-ordered). In this matrix, each parameter has a pattern of correlation coefficients with the other 5 parameters. Based on these patterns of correlation coefficients, the parameters were re-ordered by hierarchical clustering resulting in the dendrogram provided on the sides of the heatmap of Figure 7. This clustering is mainly influenced by the maximum values of the correlation coefficients of the two main correlating variables, but also takes into account the patterns of correlation coefficients with the other parameters.

9. Movie of density plots from river water experiment

As was mentioned in section 3.2 of the main manuscript, a movie of 187 density plots (i.e. 48 h in 15-min intervals) can be found as supplementary information.
